# Epithelial-myoepithelial carcinoma of the parotid gland, unusual malignancy radiologically simulating a benign lesion: case report

**DOI:** 10.1186/1477-7800-4-25

**Published:** 2007-10-16

**Authors:** Irene Piscioli, Luca Morelli, Andrea Falzone, Franca Del Nonno, Marinella Neri, Zorika Christiana Di Rocco, Alessia Catalucci, Salvatore Donato, Stefano Licci

**Affiliations:** 1 Department of Radiology, Civil Hospital of Budrio, Italy; 2 Department of Pathology, "S. Maria del Carmine" Hospital, Rovereto, Italy; 3 Department of Radiology, "S. Maria del Carmine" Hospital, Rovereto, Italy; 4 Department of Pathology, National Institute for Infectious Diseases "L. Spallanzani" IRCCS, Rome, Italy; 5 Department of Oncology, "Istituto Dermopatico dell'Immacolata", Rome, Italy; 6 Department of Radiology, "S. Salvatore" Hospital, L'aquila, Italy; 7 Department of Radiology, Civil Hospital of Bentivoglio, Italy

## Abstract

**Background:**

Ultrasound (US), Computed Tomography (CT) and Magnetic Resonance Imaging (MRI) are widely used in the clinical diagnosis of parotid gland tumors and their efficacy in identifying benign lesions is well documented. However, problems arise when facing some malignant lesions. Only few cases of salivary gland low grade malignant tumors have been previously reported in the literature complete with the radiological features.

**Case presentation:**

We here describe a case of epithelial-myoepithelial carcinoma (EMC) of the parotid gland, a low grade malignant tumor, with spread to an intraparotid lymph node and with CT and MRI findings mimicking a benign lesion.

**Conclusion:**

All the images revealed sharply outlined profiles and a homogeneous enhancement of the nodule, suggesting a benign tumor and demonstrating that a radiological evaluation of the lesion alone may be unsatisfactory and misleading in the diagnosis of salivary gland tumours, especially in the case of low grade malignant tumors, such as EMC.

## Background

Nowadays different imaging techniques offer optimal visualization of the parenchyma and of the tumors of the parotid gland. Ultrasound (US) imaging is the first-line technique in the radiological study of parotid lesions because it is an easy, quick, repeatable and non-invasive procedure. Moreover, since the parotid gland is superficial and easily accessible to the ultrasound transducer (even up to 12 MHz [1]), US is highly sensitive. However, US can be unsatisfactory in the differential diagnosis of some tumors and inflammatory diseases and it is not adequate for all glandular areas. In fact, for tumors situated in the deep lobe, which is poorly visualized with ultrasound, as it is obscured by the mandible, it is impossible to evaluate the lesions.

Contrast-enhanced computed tomography (CT) is the second choice radiological approach: it allows both assessment of the lesion and adequate exploration of the deeper gland parenchyma and offers highly defined spatial resolution along with a panoramic overview of the entire head-neck region.

Magnetic Resonance Imaging (MRI) is mandatory, representing a valid alternative to CT or a complementary approach to better assess the lesion. Compared to CT, MRI offers a much higher contrast resolution, but it is inadequate to evaluate the presence of calcifications.

CT and MRI can usually assume the benignancy or malignancy of a focal lesion based on simple elements of radiological findings. Benign lesions are usually small in size, well circumscribed, and have a regular morphology, as in the case of pleomorphic adenoma and Warthin's tumour. The characteristic radiological features of these lesions are represented by well defined margins, the presence of a capsule, and by bright signal intensity on T2-weighted MR images. Intralesional calcifications, easily detected by US and CT, are another element of benignancy.

Malignant tumors are usually medium to large in size (over 3 cm in diameter), present undefined margins and a typical heterogeneous echo pattern because of the frequent presence of necrotic areas. MRI can show multiple round high signal intensity areas on T2WI or a large very high signal intensity area on T1WI.

Epithelial-myoepithelial carcinoma (EMC) is extremely rare compared to other carcinomas, and is considered a low malignancy neoplasm.

In the present report we describe a case of this unusual parotid gland neoplasia, radiologically simulating a benign lesion.

## Case presentation

In December 2005, an 81-year-old woman came to the attention of the othorinolaryngology surgeon complaining of a painless, nodular, firm mass of the left parotid gland arisen a few months before. The patient underwent a fine needle aspiration cytology (FNAC) and 3 samples were sent to Department of Pathology. Although the CT scans suggested a benign lesion, the finding of atypical cells indicated a malignant tumor. Thus, the patient underwent a total parotidectomy with a complete excision of the lesion.

**Figure 1:**
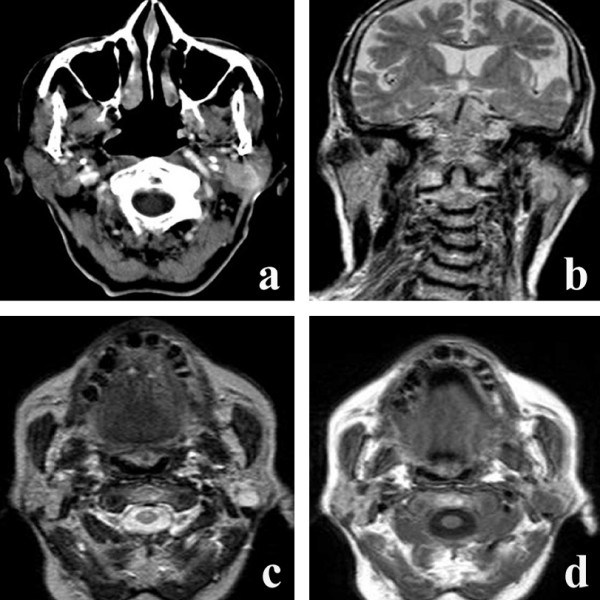
**a) **Axial contrast-enhanced CT scan shows a well-defined, oval, smooth mass in the deep portion of the left parotid gland, confined in the glandular parenchyma and enhancing. The parotid gland displays a normal morfo-volumetric aspect; **b) **Coronal T2-weighted MR image. The mass has a homogeneous moderately high signal intensity; **c) **Axial T2-weighted MR image. The lesion shows well-defined nodular mass with a moderately high signal intensity; **d) **Axial T1-weighted image of the lesion. It shows a uniform low signal intensity.

Axial contrast-enhanced CT scan (Fig. 1a) showed a well-defined, oval, smooth mass in the depth of the left parotid gland, confined to the glandular parenchyma, slight enhancing and localized in proximity to the posterior facial vein and external carotid artery, with no sign of infiltration. The parotid gland displayed a normal morfo-volumetric aspect. Coronal T2-weighted (Fig. 1b), axial T2-weighted (Fig. 1c) and axial T1-weighted (Fig. 1d) images showed a well-defined nodular mass with a moderately high signal intensity in T2-weighted images (Fig. 1b–1c), and a homogeneous low signal intensity in T1-weighted images (Fig. 1d).

All the images revealed sharply outlined profiles, suggesting a benign lesion.

Grossly, the parotid gland contained a well circumscribed gray-white nodule, partially plurinodular in structure, firm on the cut surface and 2.2 cm in diameter.

**Figure 2:**
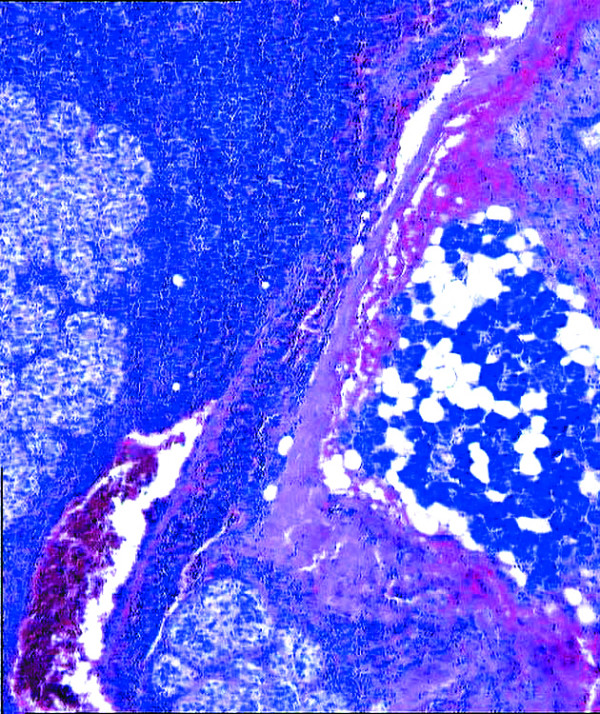
Parotid gland with the neoplasia and intraparotid lymph node involvement by the neoplastic proliferation (hematoxylin and eosin, 200×).

Histologically, the tumor was characterized by a biphasic cell population represented by myoepithelial and ductal cells. The myoepithelial cells were large, polygonal, with clear-staining cytoplasm and irregularly shaped nuclei. The ductal elements were composed of cuboidal cells with eosinophilic cytoplasm and uniform, round voluminous nuclei. The cells appeared to grow in sheets with an organoid pattern. Atypia was mild or absent. Mitotic figure count was very low. Moreover, spread to an intraparotid lymph node was observed (Fig. 2). A diagnosis of EMC was made.

US, CT and MRI are widely used in the diagnosis of parotid gland tumors. In particular CT and MRI are useful in the detection of salivary gland tumors and in assessing their extent [2]. Irregular tumor margins and infiltration into adjacent structures suggest malignancy. However, as for other human tumors, only histology can definitively establish the real nature of a lesion, given that some malignant tumors may mimic benign lesions at CT and MRI scans [3-5].

Salivary gland tumors are 12-times more frequent in the parotid gland than in the submaxillary gland, a difference that can not be explained only on the basis of gland size. Most parotid gland lesions are benign (83%) and are largely represented by mixed-type tumors [6]. CT and MRI imaging are well characterized for the latter lesions and have also been described in the following malignant parotid tumors: carcinosarcoma [7], mucoepidermoid carcinoma, adenoid cystic carcinoma, carcinoma ex pleomorphic adenoma, ductal carcinoma, myoepithelial carcinoma, sebaceous carcinoma and lymphoma [8-10].

Initially reported by Donath et al. [11], EMC arises predominantly in the parotid gland, more rarely in the submandibular and minor salivary glands.

To our knowledge, diagnostic imaging findings of EMC have been previously reported only few times [12-14].

In the report by Witterick et al. [12], the management of the lesion appears quite unusual. The right parotid mass was examined by US imaging in a follow-up period during which the size remained the same. Tumor resection was performed after a FNAC revealed malignant cells. Even though parotid EMC is a low grade carcinoma with a very slow growth, the absolutely imperceptible tumor enlargement described in the report seems to be contradicting with the intrinsic malignant nature of the lesion.

In the report by Silvers et al. [13], a case of EMC is radiologically described: CT scan showed a fairly well defined heterogeneous lesion with smooth margins and slight enhancement. MR images showed a lesion with intermediate T1-weighted signal intensity and a relatively high T2-weighted signal intensity.

More recently, Tas et al. [14] reported a case of EMC with MRI showing an irregular and heterogeneous mass, as typically seen in other more common parotid malignant tumors.

## Conclusion

In our case of parotid EMC all the images revealed sharply outlined profiles and a slight enhancement of the nodule, suggesting a benign tumor and demonstrating that a radiological evaluation of the lesion alone may be unsatisfactory and misleading in the diagnosis of salivary gland tumours, especially in the case of low grade malignant tumors, such as EMC. A FNAC of the lesion is always recommendable.

## Competing interests

The author(s) declare that they have no competing interests.

## Authors' contributions

AF, MN, AC, SD and IP participated equally in the design of the paper and in the study of the radiological data. LM, SL and FDN participated in the study of macroscopic and microscopic features of the lesion, in the design of the study and in the drafting of the manuscript. ZCDR was involved in the drafting of the manuscript. SL revised critically the final version of the manuscript. All authors read and approved the final manuscript.
